# Potential value of red blood cell distribution width in predicting in-hospital mortality in intensive care US population with acute pancreatitis: a propensity score matching analysis

**DOI:** 10.1038/s41598-023-40192-8

**Published:** 2023-08-08

**Authors:** Shaoya He, Yu Shao, Tianyang Hu, Yanan Liu

**Affiliations:** 1 Department of Gastroenterology, Anyue County People’s Hospital, Sichuan, China; 2 Department of Gastroenterology, Chongqing Emergency Medical Center, Chongqing University Central Hospital, Chongqing, China; 3 Precision Medicine Center, The Second Affiliated Hospital of Chongqing Medical University, Chongqing, China; 4 Present Address: Department of Nephrology, Rheumatology and Immunology, Jiulongpo District People’s Hospital, #23 Qianjin Road, Jiulongpo district, Chongqing, 400050 China

**Keywords:** Risk factors, Biomarkers, Outcomes research

## Abstract

The association between red blood cell distribution width (RDW) and in-hospital mortality in intensive care patients with acute pancreatitis (AP) is inconclusive. We extracted the baseline data, Bedside Index for Severity in Acute Pancreatitis (BISAP) score, Sequential Organ Failure Assessment (SOFA) score, and in-hospital prognosis of intensive care patients with AP from the Medical Information Mart for Intensive Care IV database. Performing binary logistic regression analysis to determine whether RDW is an independent risk factor for in-hospital mortality. By drawing receiver operating characteristic (ROC) curves and comparing the areas under the ROC curves (AUC) to determine the predictive value of RDW for in-hospital mortality, and by conducting survival analysis to evaluate the impact of RDW on survival time in hospital. Before and after the propensity score matching (PSM) analysis, RDW was always a risk factor for in-hospital mortality in patients with AP. The AUC of RDW was comparable to BISAP, while the AUCs of combining RDW and BISAP or SOFA were greater than that of BISAP or SOFA alone. The median survival time of the high-RDW group (RDW > 15.37%, before PSM; RDW > 15.35%, after PSM) was shorter than that of the low-RDW group. Compared with the low-RDW group, the hazard ratios of the high-RDW group were 3.0708 (before PSM) and 1.4197 (after PSM). RDW is an independent risk factor for in-hospital mortality in patients with AP. The predictive value of RDW for in-hospital mortality of patients with AP is comparable to BISAP, and the combination of RDW and BISAP or SOFA scoring system can improve the predictive performance to a certain extent.

## Introduction

Acute pancreatitis (AP) is a pancreatic inflammatory disorder that manifests as acinar cell death, local and systemic inflammation^1^. The global incidence of AP is 34 individuals per 100,000 person-years, and the incidence has been increasing^2^. Although the mortality associated with AP has decreased from 1.6 to 0.8% in the past decade, it is still far from satisfactory. Early identification of patients at high risk of death and early intervention is still an important clinical issue.

Red blood cell distribution width (RDW) is an item of the routine complete blood count, which has been confirmed to be related to AP by several studies. Uçar Karabulut et al.^3^ compared the RDW values of 104 patients with AP and found that the values of samples collected during hospitalization were significantly higher than that of samples after full recovery (*p* < 0.05). They believed that RDW can be used as a tool for early diagnosis and assessment of the progression of AP. Wang et al.^4^ investigated the relationship between RDW and the severity of AP. 527 patients were grouped according to the severity (mild AP group, moderately severe AP group, and severe AP group), and 105 non-AP patients during the same period were used as controls. It was found that RDW was significantly higher in severe AP patients than in mild AP and moderately severe AP patients (*P* < 0.05). In addition, Zhang et al.^5^ investigated the relationship between RDW and the 90-day mortality of severe AP and found that the values of RDW were greater in the non-surviving severe AP patients than in the surviving patients.

In general, there are currently few studies on the relationship between RDW and AP, and the sample of cases included in related studies is slightly insufficient and the quality of evidence is low. Meanwhile, there have been no studies directly investigating the relationship between RDW and AP in intensive care. In addition, the study by Uçar Karabulut et al.^3^ showed that there are significant differences in the RDW values during the onset and recovery period of AP, indicating that the RDW value changes dynamically with the progression of the disease. Then, it may be inappropriate to predict mid- to long-term mortality based on a single RDW value at a certain time node. This study aims to explore the association between RDW and in-hospital mortality of intensive care patients with AP with a larger sample of cases based on the well-known public medical database Medical Information Mart for Intensive Care-IV (MIMIC-IV, https://mimic.mit.edu/).

## Methods

### Database

The MIMIC-IV database contains health-related data from patients in the intensive care unit (ICU) of the Beth Israel Deaconess Medical Center from 2008 to 2019. Before conducting medical research based on the MIMIC-IV database, researchers need to obtain access rights and need to participate in and pass the "Protecting Human Research Participants" exam on the National Institutes of Health website. The author Tianyang Hu passed the above exam (Certification No. 37474354) and was allowed to access the database and extract data. This study was ethically approved by an affiliate of the Massachusetts Institute of Technology (No. 27653720). All patient-related information in the database is anonymous, and informed consent was obtained from all subjects and/or their legal guardian(s). The study complies with the provisions of the Declaration of Helsinki.

### Study population and data extraction

The inclusion criteria for this study were: patients admitted to the ICU and diagnosed with acute pancreatitis. Exclusion criteria include: patients younger than 18 years old; patients with missing RDW values within 24 h of admission. In this study, the BISAP score and SOFA score were used as references to evaluate the value of RDW. We extracted the following variables of the patient: age, gender, length of hospital stay, length of ICU stay, hospital expire flag (a binary variable indicating whether the patient died in the hospital), laboratory measurements (including RDW, red blood cell, white blood cell, platelets, anion gap, creatinine, and international normalized ratio), vital signs (including heart rate, systolic blood pressure, diastolic blood pressure, respiratory rate, and body temperature), Charlson comorbidity index, Bedside Index for Severity in Acute Pancreatitis (BISAP) score, and Sequential Organ Failure Assessment (SOFA) score. Laboratory measurements, vital signs, and scoring system scores were collected in the first 24 h of admission, and if a variable was measured multiple times, the average was taken.

### Statistical analysis

All statistical analysis was performed by Medcalc software (version 19.6.1). A *P* value < 0.05 was regarded as statistically significant. The normality of continuous variables was evaluated by the Kolmogorov–Smirnov test, the variables that obey the normal distribution were expressed as mean ± standard deviation (M ± SD), and the independent sample t-test was used for comparison between groups; the variables that do not obey the normal distribution were expressed as the median with interquartile range (IQR), and the Wilcoxon rank-sum was used for comparison. Categorical variables were expressed as numbers and percentages, and the Chi-square test was used for comparison.

To minimize the selection bias in this retrospective study, we performed a propensity score matching (PSM) analysis to control the differences between the survival group and the death group. Patients from the two groups were paired by a 1:1 nearest neighbor matching algorithm (a caliper of 0.02) based on the three variables: age, gender, and Charlson comorbidity index (a weighted index^6^ that takes into account the number and the seriousness of comorbidities, including myocardial infarct, congestive heart failure, peripheral vascular disease, cerebrovascular disease, dementia, chronic pulmonary disease, rheumatic disease, peptic ulcer disease, liver disease, diabetes, malignant cancer, acquired immune deficiency syndrome, etc.), and the propensity score was calculated by a logistic regression model. To determine whether RDW is an independent risk factor for in-hospital mortality of intensive care patients with AP, we conducted the binary logistic regression analysis. Variables with *P* values less than 0.1 in the univariate analysis were included in the multivariate analysis. To clarify the predictive value of RDW for in-hospital mortality of intensive care patients with AP, we compared the area under the receiver operating characteristic (ROC) curve (AUC) of RDW, BISAP, SOFA, RDW combined with BISAP (RDW + BISAP), and RDW combined with SOFA (RDW + SOFA). The Z test was performed by the method of Delong et al.^7^. To further investigate the relationship between RDW and in-hospital mortality of intensive care patients with AP, we conducted survival analysis by Kaplan–Meier curves. In-hospital mortality could be regarded as a time-to-event variable (the event is death during hospitalization). Patients were followed during the hospital stay, and patients were censored when discharged alive. RDW values were divided into the high-RDW group and low-RDW group according to the optimal cut-off values from the results of the ROC curves, and the log-rank test was performed to evaluate the difference in survival rate between the two groups.

## Results

### Baseline characteristics

**Figure 1:**
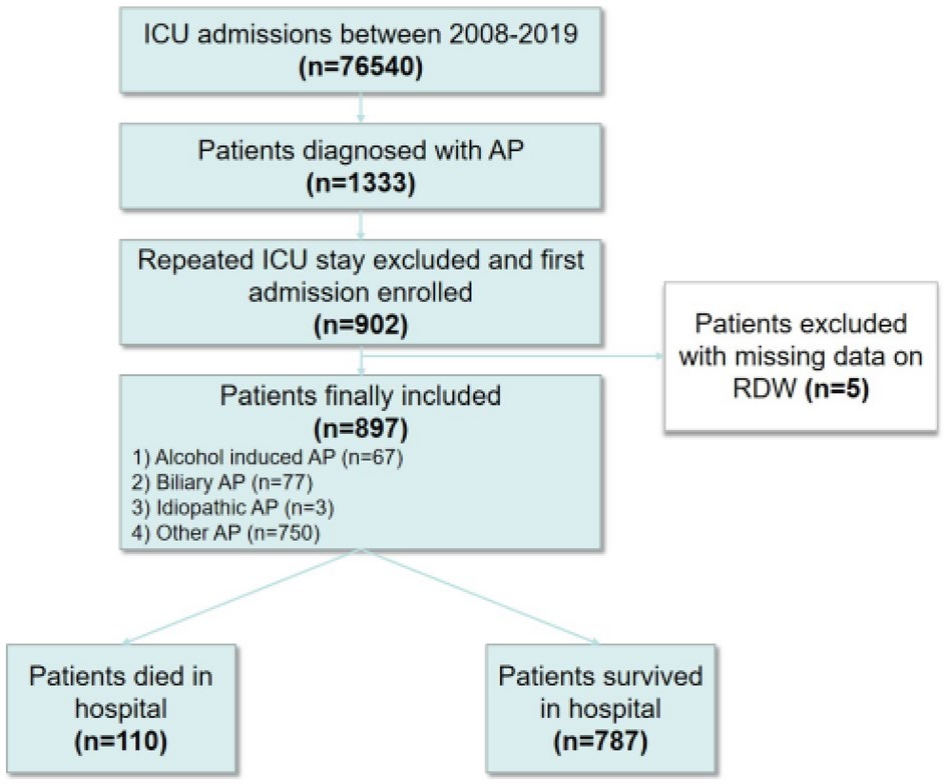
Flowchart of study cohort.

We finally included 897 patients with AP, of which 110 patients died, 787 patients survived (Fig. 1), and the in-hospital mortality rate was 12.26%. Age, Charlson comorbidity index, level of RDW, BISAP score, and SOFA score of the death group were significantly higher than those of the survival group, and the length of ICU stay of the death group was also significantly longer than that of the survival group (*P* < 0.001 for all). After PSM, there was no significant difference in age and Charlson comorbidity index between the two groups (*P* > 0.05 for all), and the gender between the two groups was completely matched (*P* = 1.000). The level of RDW, BISAP score, and SOFA score of the death group were still significantly higher than those of the survival group (*P* < 0.05 for all). All the baseline characteristics are presented in Table 1.

**Table 1:** Demographic and clinical characteristics of the study population.

Characteristics	Before PSM			After PSM		
	Death (n = 110)	Survival (n = 787)	*P*	Death (n = 107)	Survival (n = 107)	*P*
*Age (year)	67.5 (57.0–81.0)	57.0 (46.0–71.0)	< 0.001	69.5 (57.5–81.0)	70.0 (60.0–81.0)	0.836
*Gender (Male)	57 (51.8)	466 (59.2)	0.141	55 (51.4)	55 (51.4)	1.000
LOS ICU (day)	5.4 (1.6–12.8)	2.8 (1.4–5.9)	0.001	4.3 (1.5–11.4)	2.2 (1.2–4.3)	< 0.001
LOS HOS(day)	10.8 (3.4–19.5)	11.4 (6.5–20.0)	0.487	12.9 (3.5–21.5)	10.8 (5.9–19.0)	0.943
*CCI	6 (5–8)	4 (2–6)	< 0.001	6 (5–8)	6 (4–7)	0.197
Laboratory measurements						
RBC(10^12^/L)	3.4 (2.9–4.1)	3.7 (3.2–4.2)	< 0.001	3.4 (2.8–4.1)	3.7 (3.2–4.2)	0.010
WBC(10^9^/L)	14.6 (9.3–21.0)	12.4 (8.6–17.4)	0.007	14.1 (9.4–19.6)	12.9 (8.8–19.8)	0.197
PLT(10^9^/L)	146 (93–239)	190 (137–265)	0.002	152 (96–250)	193 (144–280)	0.017
AG(mmol/L)	18.0 (14.5–22.0)	15.0 (13.0–17.5)	< 0.001	17.5 (14.0–21.3)	15.0 (13.0–16.5)	< 0.001
Cr(ng/dL)	1.8 (1.2–3.3)	1.0 (0.7–1.7)	< 0.001	1.7 (1.1–3.0)	1.1 (0.8–1.6)	< 0.001
INR	1.5 (1.3–2.1)	1.3 (1.2–1.5)	< 0.001	1.5 (1.3–2.0)	1.4 (1.2–1.6)	0.002
Vital signs						
HR(bpm)	93 (86–109)	95 (81–108)	0.290	95 (87–110)	91 (78–106)	0.029
SBP (mmHg)	108 (100–119)	123 (111–137)	< 0.001	108 (100–119)	125 (110–139)	< 0.001
DBP (mmHg)	59 (53–67)	69 (61–78)	< 0.001	59 (53–68)	67 (56–74)	0.012
RR(cpm)	22 (19–25)	20 (17–23)	< 0.001	22 (20–25)	20 (18–22)	< 0.001
T(℃)	36.8 (36.4–37.2)	37.0 (36.7–37.4)	< 0.001	36.8 (36.5–37.2)	36.8 (36.6–37.1)	0.184
RDW (%)	15.75 (14.43–17.45)	14.54 (13.60–15.69)	< 0.001	15.67 (14.43–17.25)	14.87 (13.95–15.75)	< 0.001
BISAP score	3 (3–4)	2 (2–3)	< 0.001	3 (3–4)	3 (2–4)	0.001
SOFA score	12 (8–15)	5 (3–8)	< 0.001	11 (7–14)	5 (3–7)	< 0.001

Values are expressed as the median (IQR) or n (%).

*PSM* propensity score matching, *LOS ICU* length of intensive care unit stay, *LOS HOS* length of hospital stay, *CCI* Charlson Comorbidity Index, *RBC* red blood cell, WBC white blood cell, *PLT* platelets, *AG* anion gap, *Cr* creatinine, *INR* international normalized ratio, *HR* heart rate, bpm beat per minute, *SBP* systolic blood pressure, DBP diastolic blood pressure, *RR* respiratory rate, *cpm* count per minute, *T* temperature, *RDW* red blood cell distribution width, *BISAP* bedside index for severity in acute pancreatitis, *SOFA* sequential organ failure assessment.

*Covariables included in the PSM.

### Logistic regression analysis

Before PSM, RDW was an independent risk factor for in-hospital mortality in patients with AP before (OR: 1.347, 95% CI: 1.233–1.471, *P* < 0.001) and after (OR: 1.265, 95% CI: 1.124–1.423, *P* < 0.001) adjustment (Table 2). After PSM, RDW was still an independent risk factor for in-hospital mortality in patients with AP before (OR: 1.400, 95% CI: 1.188–1.649, *P* < 0.001) and after (OR: 1.262, 95% CI: 1.033–1.591, *P* = 0.024) adjustment, while respiratory rate was also an independent risk factor for in-hospital mortality in patients with AP (after PSM, OR: 1.200, 95% CI: 1.065–1.351, *P* = 0.003) (Table 3).

**Table 2:** Binomial Logistic regression analysis (before PSM).

Variable	Univariable		Multivariable	
	OR (95% CI)	*P*	OR (95% CI)	*P*
Age	1.038 (1.025–1.051)	< 0.001	1.026 (1.002–1.051)	0.033
Gender	0.741 (0.497–1.105)	0.142		
LOS ICU	1.023 (1.006–1.041)	0.007	0.985 (0.956–1.015)	0.322
LOS HOS	1.001 (0.989–1.013)	0.829		
CCI	1.289 (1.203–1.380)	< 0.001	1.193 (1.073–1.326)	0.001
Red blood cell	0.619 (0.468–0.820)	0.001	0.854 (0.598–1.218)	0.383
WBC	1.045 (1.022–1.069)	< 0.001	1.037 (1.010–1.065)	0.007
Platelets	0.997 (0.996–0.999)	0.012	1.000 (0.998–1.002)	0.897
Anion gap	1.108 (1.069–1.149)	< 0.001	1.011 (0.953–1.072)	0.727
Creatinine	1.216 (1.115–1.327)	< 0.001	0.970 (0.824–1.142)	0.715
INR	1.282 (1.065–1.543)	0.009	0.753 (0.545–1.041)	0.086
Heart rate	1.005 (0.995–1.016)	0.332		
SBP	0.956 (0.943–0.970)	< 0.001	0.980 (0.960–1.000)	0.055
DBP	0.951 (0.934–0.969)	< 0.001	1.018 (0.987–1.051)	0.258
RR	1.103 (1.055–1.153)	< 0.001	1.127 (1.058–1.200)	< 0.001
Temperature	0.425 (0.303–0.597)	< 0.001	0.529 (0.352–0.795)	0.002
RDW	1.347 (1.233–1.471)	< 0.001	1.265 (1.124–1.423)	< 0.001
BISAP score	2.503 (1.975–3.171)	< 0.001	1.157 (0.795–1.686)	0.446
SOFA score	1.240 (1.187–1.296)	< 0.001	1.221 (1.125–1.324)	< 0.001

*PSM* propensity score matching, *OR* odds ratio, *CI* confidence interval, *LOS ICU* length of intensive care unit stay, *LOS HOS* length of hospital stay, *CCI* Charlson Comorbidity Index, *WBC* white blood cell, *INR* international normalized ratio, *SBP* systolic blood pressure, *DBP* diastolic blood pressure, *RR* respiratory rate, *RDW* red blood cell distribution width, *BISAP* bedside index for severity in acute pancreatitis, *SOFA* sequential organ failure assessment.

**Table 3:** Binomial Logistic regression analysis (after PSM).

Variable	Univariable		Multivariable	
	OR (95% CI)	*P*	OR (95% CI)	*P*
Age	0.997 (0.979–1.014)	0.716		
Gender	1.000 (0.585–1.709)	1.000		
LOS ICU	1.085 (1.036–1.137)	0.001	1.015 (0.959–1.074)	0.603
LOS HOS	1.015 (0.995–1.035)	0.133		
CCI	1.061 (0.948–1.189)	0.303		
Red blood cell	0.657 (0.458–0.942)	0.022	0.844 (0.483–1.474)	0.551
WBC	1.024 (0.995–1.055)	0.105		
Platelets	0.998 (0.995–1.000)	0.031	1.000 (0.997–1.003)	0.769
Anion gap	1.141 (1.068–1.218)	< 0.001	0.951 (0.862–1.049)	0.311
Creatinine	1.767 (1.340–2.331)	< 0.001	1.340 (0.911–1.972)	0.137
INR	1.703 (1.072–2.706)	0.024	0.712 (0.387–1.310)	0.275
Heart rate	1.019 (1.003–1.036)	0.024	1.026 (1.000–1.052)	0.053
SBP	0.964 (0.948–0.979)	< 0.001	0.990 (0.961–1.020)	0.507
DBP	0.977 (0.956–0.999)	0.038	1.009 (0.965–1.055)	0.689
RR	1.203 (1.112–1.301)	< 0.001	1.200 (1.065–1.351)	0.003
Temperature	0.656 (0.432–0.997)	0.048	0.603 (0.309–1.179)	0.139
RDW	1.400 (1.188–1.649)	< 0.001	1.262 (1.033–1.591)	0.024
BISAP score	1.637 (1.223–2.191)	0.001	1.002 (0.639–1.571)	0.994
SOFA score	1.335 (1.230–1.449)	< 0.001	1.255 (1.102–1.430)	0.001

*PSM* propensity score matching, *OR* odds ratio, *CI* confidence interval, *LOS ICU* length of intensive care unit stay, *LOS HOS* length of hospital stay, *CCI* Charlson Comorbidity Index, *WBC* white blood cell, *INR* international normalized ratio, *SBP* systolic blood pressure, *DBP* diastolic blood pressure, *RR* respiratory rate, *RDW* red blood cell distribution width, *BISAP* bedside index for severity in acute pancreatitis, *SOFA* sequential organ failure assessment.

### Comparison of ROC curves

**Figure 2:**
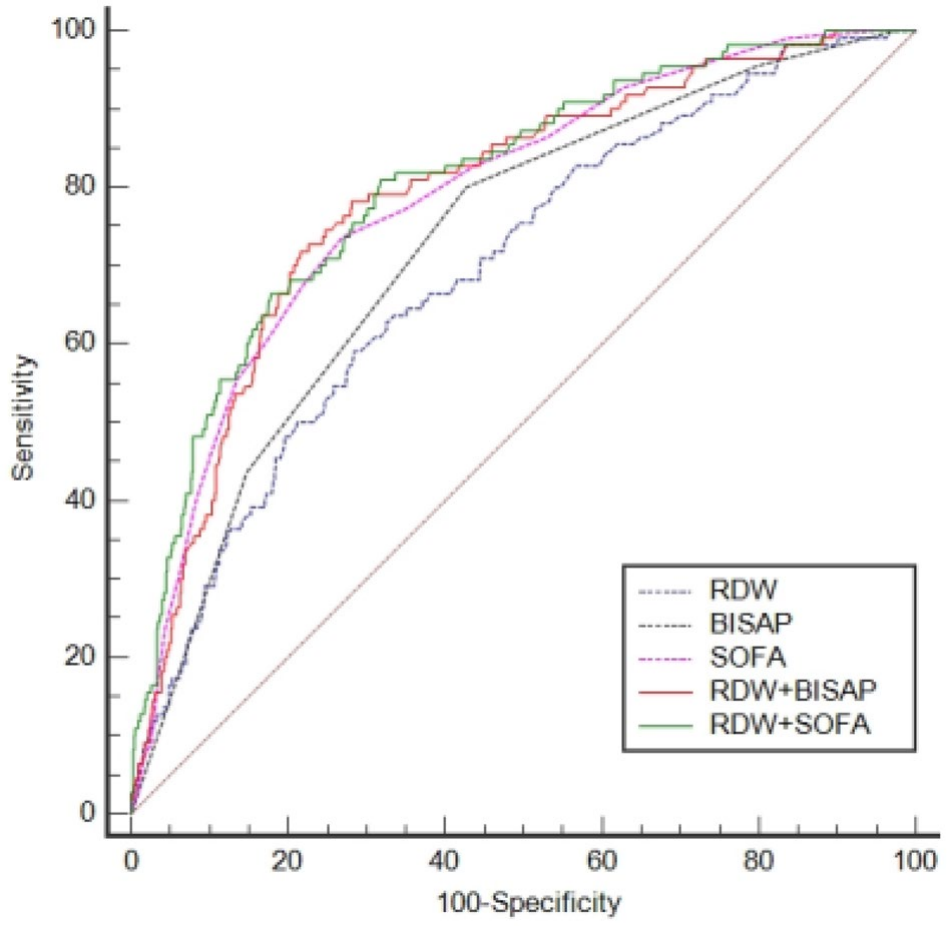
ROC curves of RDW, BISAP, SOFA, RDW + BISAP, RDW + SOFA (before propensity score matching).

**Figure 3:**
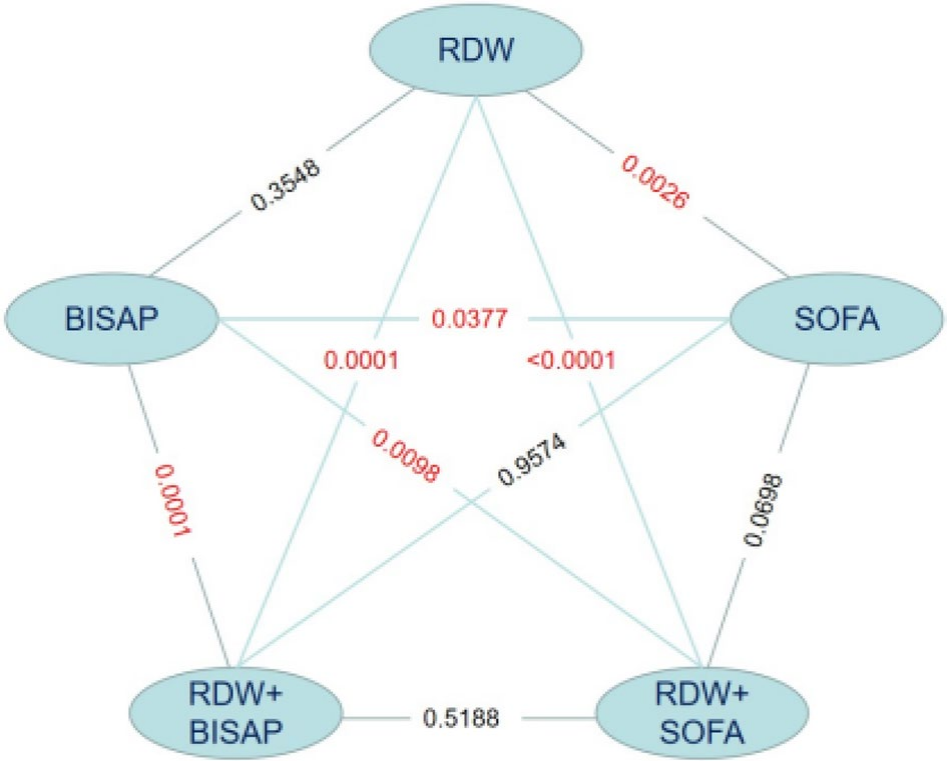
Comparison of Z tests (before propensity score matching).

Before PSM, the AUCs of RDW, BISAP, SOFA, RDW + BISAP, and RDW + SOFA were 0.696, 0.729, 0.788, 0.790, and 0.805, respectively (Fig. 2, Table 4). The predictive value of RDW was comparable to that of BISAP (*P* = 0.3548), and the predictive value of RDW + BISAP was significantly higher than that of BISAP (P = 0.0001). The predictive value of RDW + BISAP was equivalent to RDW + SOFA (P = 0.5188), and RDW + BISAP had the highest Youden’s index of 0.5022 (Fig. 3, Table 4).

**Table 4:** Comparison of ROC curves (before PSM).

Factor	AUC	95%CI	Optimal cut-off	Sensitivity (%)	Specificity (%)	Youden’s index
RDW	0.696	0.665–0.726	15.37%	59.09	71.54	0.3063
BISAP	0.729	0.698–0.757	2	80.00	57.31	0.3731
SOFA	0.788	0.760–0.815	7	73.64	73.06	0.4670
RDW + BISAP	0.790	0.762–0.816	0.16181^#^	71.82	78.40	0.5022
RDW + SOFA	0.805	0.778–0.831	0.09119*	80.91	68.23	0.4914

*RDW* red blood cell distribution width, *BISAP* bedside index for severity in acute pancreatitis, *SOFA* sequential organ failure assessment.

^#^Prediction probability of logistic regression model for combining RDW and BISAP, corresponding to RDW = 15.95%, BISAP = 3.

*Prediction probability of logistic regression model for combining RDW and SOFA, corresponding to RDW = 14.95%, SOFA = 7.

**Figure 4:**
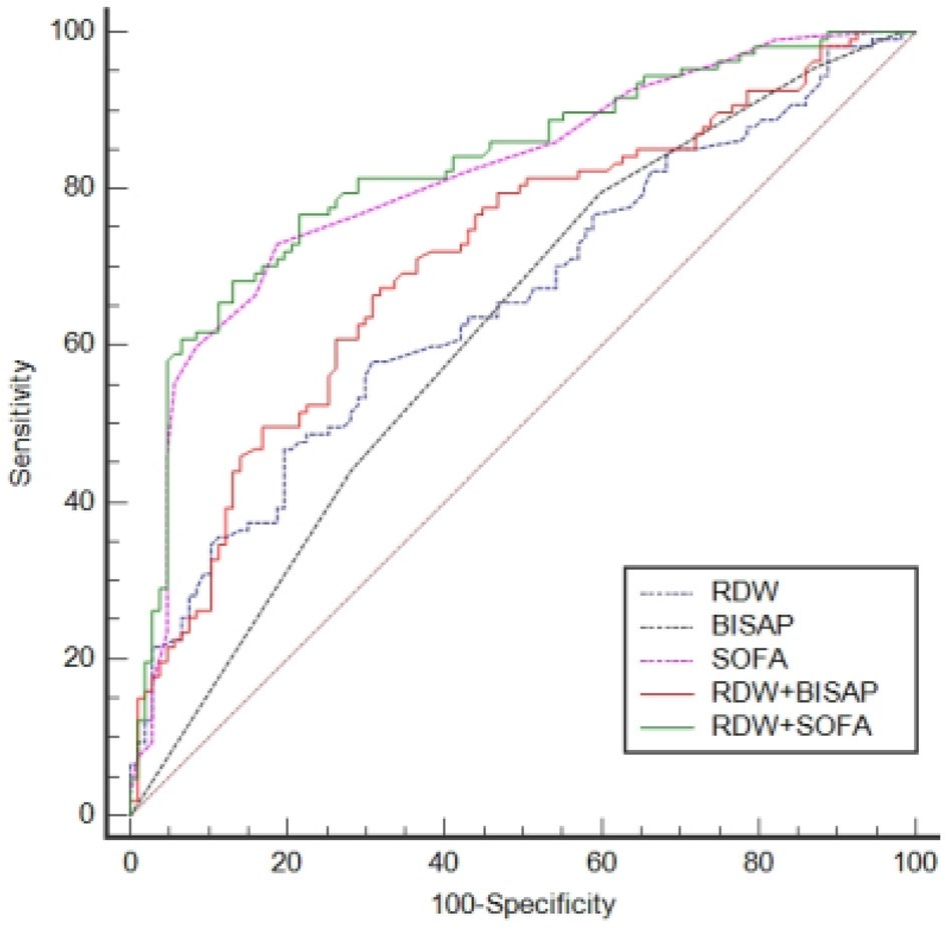
ROC curves of RDW, BISAP, SOFA, RDW + BISAP, RDW + SOFA (after propensity score matching).

**Figure 5:**
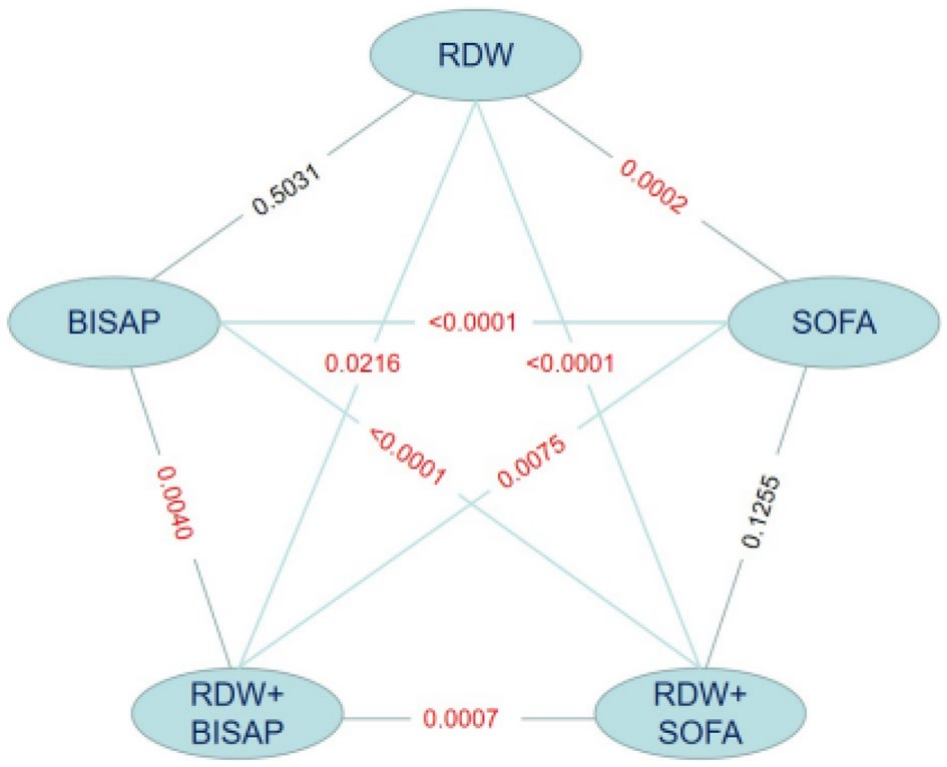
Comparison of Z tests (after propensity score matching).

After PSM, the AUCs of RDW, BISAP, SOFA, RDW + BISAP, and RDW + SOFA were 0.656, 0.623, 0.815, 0.713, and 0.827, respectively (Fig. 4, Table 5). The predictive value of RDW was still comparable to that of BISAP (*P* = 0.5031), and the predictive value of RDW + BISAP was significantly higher than that of BISAP (*P* = 0.0040). The predicted value of RDW + SOFA was higher than RDW + BISAP (*P* = 0.0007), and RDW + SOFA had the highest Youden’s index of 0.5514 (Fig. 5, Table 5).

**Table 5:** Comparison of ROC curves (after PSM).

Factor	AUC	95%CI	Optimal cut-off	Sensitivity (%)	Specificity (%)	Youden’s index
RDW	0.656	0.588–0.719	15.35%	57.94	69.16	0.2710
BISAP	0.623	0.554–0.688	2	79.44	40.19	0.1963
SOFA	0.815	0.756–0.865	7	72.90	81.31	0.5421
RDW + BISAP	0.713	0.647–0.772	0.50287^#^	67.29	68.22	0.3551
RDW + SOFA	0.827	0.770–0.875	0.46916*	76.64	78.50	0.5514

*RDW* red blood cell distribution width, *BISAP* bedside index for severity in acute pancreatitis, *SOFA* sequential organ failure assessment.

^#^Prediction probability of logistic regression model for combining RDW and BISAP, corresponding to RDW = 14.05%, BISAP = 4.

*Prediction probability of logistic regression model for combining RDW and SOFA, corresponding to RDW = 14.40%, SOFA = 8.

### Comparison of Kaplan–Meier curves

**Figure 6:**
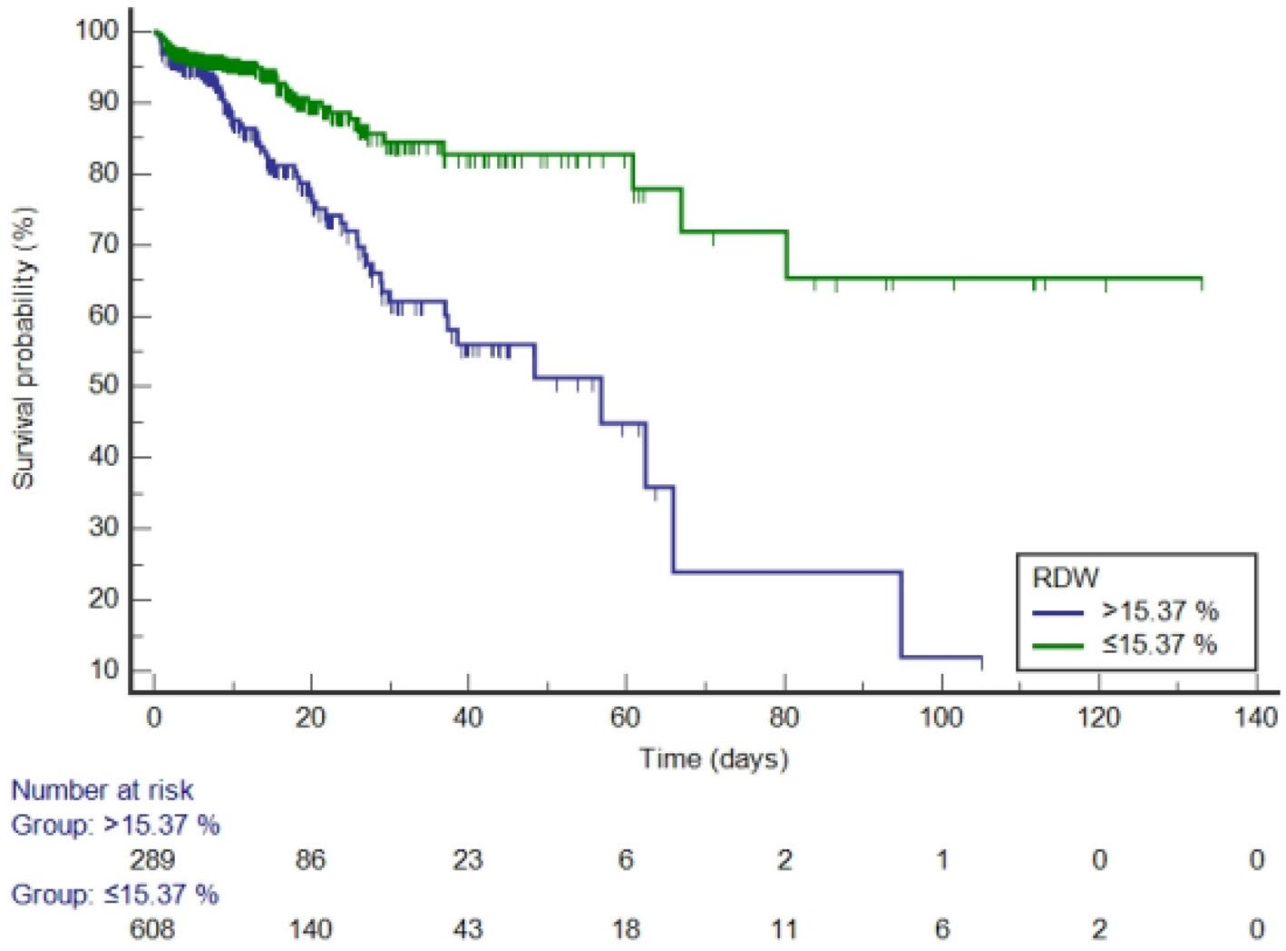
Kaplan–Meier survival curves by RDW category (before propensity score matching, log-rank *P* < 0.0001).

RDW values were divided into high-RDW group and low-RDW group according to the optimal cut-off value of 15.37% before PSM (Fig. 6). The median survival time of the high-RDW group was 56.770 days (95% CI: 36.990–65.910), while of the low-RDW group was 102.345 days (95% CI: 89.696–114.993). Compared with the low-RDW group, the hazard ratio (HR) of the high-RDW group was 3.0708 (95% CI: 2.0665–4.5631, *P* < 0.0001).

**Figure 7:**
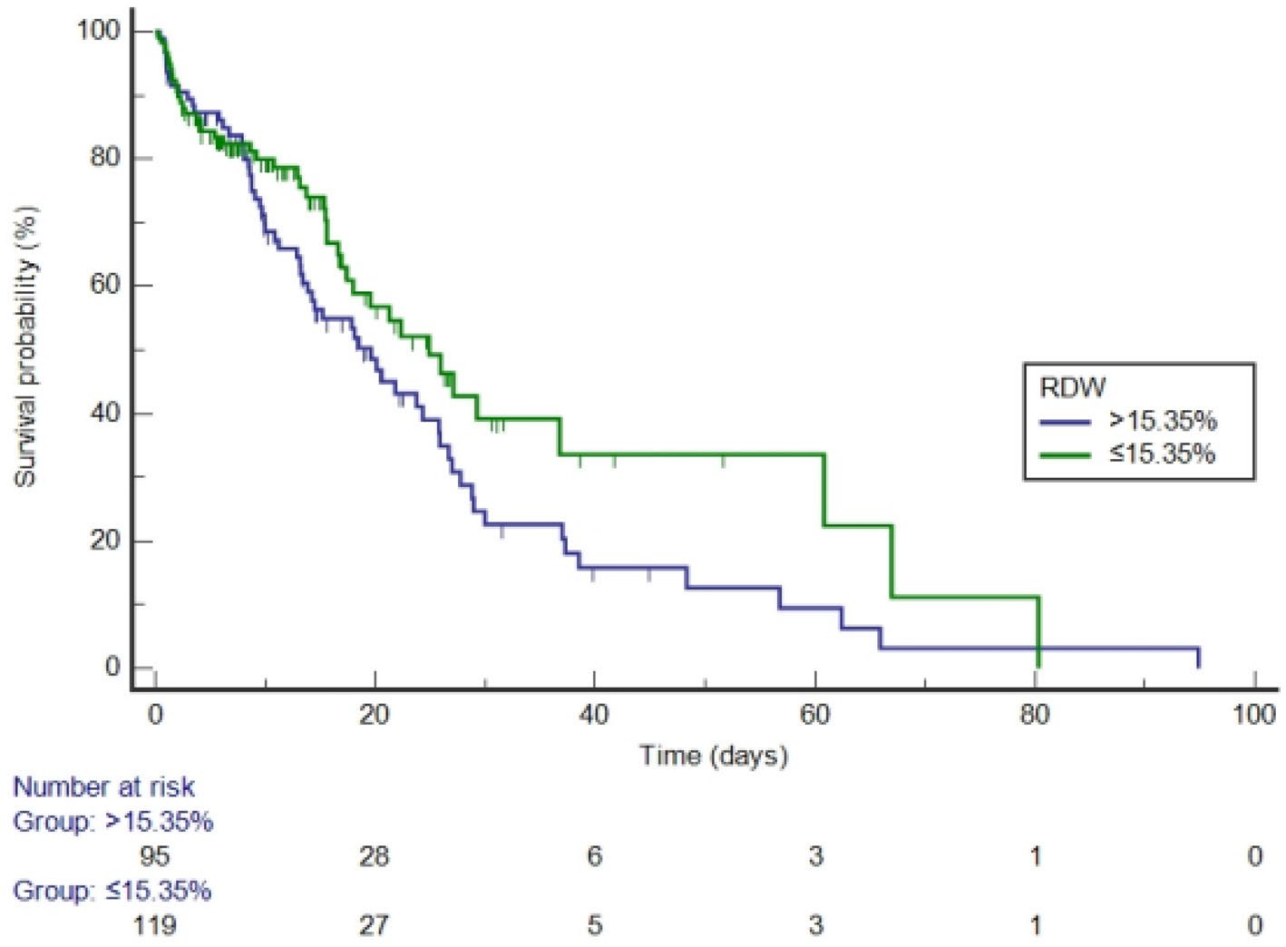
Kaplan–Meier survival curves by RDW category (after propensity score matching, log-rank *P* = 0.0721).

After PSM, RDW values were divided into two groups according to the optimal cut-off value of 15.35% (Fig. 7). The median survival time of the high-RDW group was 19.617 days (95% CI: 13.390–25.783), while of the low-RDW group was 24.887 days (95% CI: 17.400–36.756). Compared with the low-RDW group, the hazard ratio (HR) of the high-RDW group was 1.4197 (95% CI: 0.9719–2.0740, *P* = 0.0721).

## Discussion

This study investigated the association between RDW and in-hospital mortality in intensive care patients with AP and confirmed that RDW is an independent risk factor for in-hospital mortality in AP. In the binomial logistic regression analysis, the OR of RDW was greater than 1.2 for both univariable and multivariable logistic regression analyses, as well as before and after PSM, and all lower bounds were greater than 1 (all p values were less than 0.05). RDW is a parameter that reflects the heterogeneity of red blood cells, and researchers have confirmed that a high level of RDW is independently related to the poor prognosis of intensive care patients with sepsis^8–10^. However, the pathophysiology of RDW in AP has not yet been fully clarified. In the course of AP, pancreatin itself activates, releases excessive inflammatory mediators, damages endothelial cells, and further triggers a cascade of inflammatory response and oxidative stress^11,12^. RDW is associated with pro-inflammatory cytokines^13^, and the inflammatory response during AP affects hematopoietic function^14^, inhibits the maturation of red blood cells, the production and release of erythropoietin, and causes immature red blood cells to enter the blood circulation, resulting in an increase in RDW; meanwhile, RDW is also associated with oxidative stress reactions^15^, oxidative stress can also shorten the survival time of red blood cells and promote naive red blood cells to enter the blood^16^, which can also be manifested as an increase in RDW; in addition, malnutrition and hypoproteinemia associated with AP can also cause anemia^17^, leading to an increase in RDW. Therefore, the above reasons may be able to explain the association between RDW and AP. Zhou et al. also previously explored the prognostic predictive value of RDW in Chinese AP patients and found that RDW not only predicted the occurrence of severe AP, but also 28-day mortality^18^. Our study fills in the value of RDW in AP populations of western developed countries to a certain extent, because there is a difference in the pathogenesis of AP between China and developed countries, with biliary tract disease being the more than usual cause of AP in China, whereas in developed countries, such as the US, alcoholism is a major cause of AP in addition to biliary tract disease^19,20^. At the same time, the larger sample size of our study and the use of the PSM method make our conclusions more reliable. In addition, since the populations we focused on were all from ICUs, our study fills a gap in research on the value of RDW in the intensive care AP population. In addition to RDW, after PSM we also found that respiratory rate was also an independent risk factor for in-hospital mortality in the patients. Elevated respiratory rate usually indicates that patients may be comorbid with respiratory infections, heart failure, and other diseases, and these comorbidities naturally increase the mortality rate of AP patients. However, RDW is still an independent risk factor for in-hospital mortality in AP after adjusting the confounding factors such as respiratory rate.

In view of the fact that there are no effective drugs to treat AP currently, most care is supportive^21^. Thus, it is necessary to evaluate the prognosis of AP, especially the prediction of in-hospital mortality. If clinicians could rapidly identify patients at high risk of death, more aggressive therapy and care measures can help reduce mortality. In fact, currently, the predictive value of any single variable to predict mortality in patients is very low. We investigated the predictive value of RDW in in-hospital mortality of AP, and we found that the AUC for RDW in predicting in-hospital mortality in acute pancreatitis was 0.696 (before PSM) and 0.656 (after PSM), respectively, and such predictive value is already high for a single variable. The BISAP was proposed by Wu et al. in 2008 and proved to be useful for the early identification of AP patients at increased risk for in-hospital mortality^22^. BISAP is scored with the presence of the following (one point for one variable): Blood urea nitrogen > 25 mg/dl, Impaired mental state (Glasgow coma score < 15), Systemic inflammatory response syndrome (SIRS), Age > 60 years, Presence of pleural effusion. In this study, the value of BISAP in predicting in-hospital mortality of AP was unexpectedly excellent, and its AUCs were only 0.729 (before PSM) and 0.623 (after PSM). However, clinicians tend to use a simpler scoring system in clinical practice, and as a single factor, the equivalent predictive value of RDW shows great application potential. SOFA scoring system is a good indicator of prognosis during the first few days of ICU admission^23^. We combined RDW with BISAP or SOFA and found that the predictive value of the combinations was better than that of BISAP or SOFA alone, either with an increase in AUC or an increase in the Youden’s index (Tables 4, 5). In clinical practice, we can actually hardly expect to rely on a single variable to predict mortality, hence the derivation of a series of scoring systems containing multiple variables (e.g., the BISAP score here). Adding a predictor to another existing scoring system may quickly improve prediction performance. In this study, we give the example of RDW + BISAP/SOFA to show that RDW combined with other metrics could undoubtedly improve the predictive value, but RDW + BISAP/SOFA are by no means the new scoring systems we ultimately want. We believe that a scoring system built on the basis of RDW (e.g., built in the way of current mainstream machine learning algorithms, etc.) is definitely superior to RDW + BISAP/SOFA, and that RDW may have a broader application prospect in predicting the in-hospital mortality of AP.

It is generally believed that the normal range of RDW is ≤ 15%^24–26^. In the survival analysis, the cut-off values indicated by the ROC curves of RDW were 15.37% (before PSM) and 15.35% (after PSM), which were not much different from the normal cut-off value of 15%. Our study found that the median survival time of the high-RDW group was always shorter than that of the low-RDW group. Before PSM, compared with the low-RDW group, the HR of the high-RDW group was 3.0708, suggesting that the risk of in-hospital death in the high-RDW group was about three times that of the low-RDW group. After PSM, the risk of in-hospital death in the high-RDW group was 1.4197 times that of the low-RDW group. Although the difference between the two groups was not statistically significant, it can be seen from the Kaplan–Meier curves (Fig. 7) that the survival curves of the two groups blended in the first 10 days, however, after about 10 days, the survival curve of the low-RDW group was almost always above the curve of the high-RDW group. Therefore, the above results once again confirmed that a high level of RDW on the first day is significantly associated with an increase in in-hospital mortality of patients with AP.

This study has certain limitations: (1) Of the patients included in this study, 67 were diagnosed with alcohol induced AP, 77 were with biliary AP, and 3 were with idiopathic AP. The other patients had no specified cause. Therefore, we did not conduct subgroup analysis or stratified analysis at the etiology level; (2) Since the patients included in this study were from the MIMIC-IV, a critical illness database, we default that all patients were severe AP. However, the data in MIMIC-IV cannot reflect whether the condition of one patient was severe or mild. As a consequence, we did not classify the severity of the patients, and we did not consider the severity when performing the PSM. Meanwhile, the applicability of our conclusions to the general ward is something that needs further validation; (3) Dynamic monitoring of changes in laboratory tests, vital signs, and scoring system scores facilitates rapid reflection of changes in the patient's condition, but we could not normalize these variables, which could potentially affect the stability of the results. Therefore, the conclusions of this study still need to be validated by prospective studies with an adequate design that should pay particular attention to the severity of the patient's condition and the exact etiology of acute pancreatitis. If possible, dynamic changes in variables such as RDW should also be accurately recorded. It is worth mentioning that the sample size included in this study is relatively large, which makes our results more stable and reliable. Monitoring the RDW level is easy to perform and inexpensive, which is especially suitable for promotion in countries or regions with limited resources. All in all, we confirmed the association between RDW and in-hospital mortality of intensive care patients with AP, and the predictive value of RDW for in-hospital mortality of patients with AP is comparable to BISAP.

## Conclusions

RDW is an independent risk factor for in-hospital mortality in patients with AP. The predictive value of RDW for in-hospital mortality of patients with AP is comparable to BISAP, and the combination of RDW and BISAP or SOFA scoring system can improve the predictive performance to a certain extent. However, the conclusions of this study still need to be verified by prospective studies with an adequate design.

## Data Availability

The datasets used and/or analysed during the current study available from the corresponding author on reasonable request.
